# Supplementary material for: Hypoconnectivity between anterior insula and amygdala associates with future vulnerabilities in social development in a neurodiverse sample of neonates
Source: Sci Rep. 2022 Sep 28;12:16230. doi: 10.1038/s41598-022-20617-6 (PMC9517994; doi:10.1038/s41598-022-20617-6)

**Supplemental Materials**

**Figure S1:** Regions of interest used for seed connectivity. The right and left insula seeds are shown in orange and red, respectively.


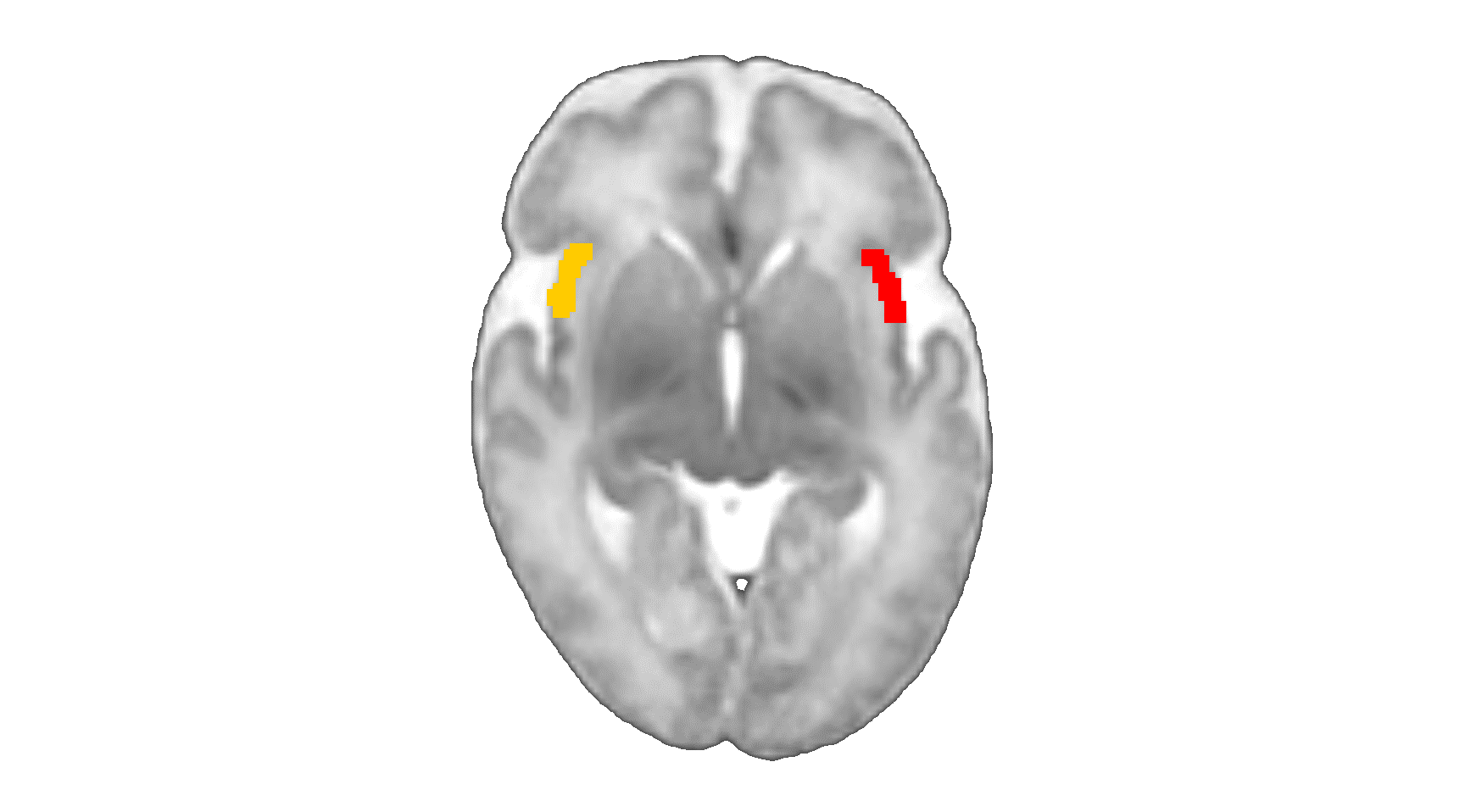


**Figure S2:** Comparison of Insula-amygdala connectivity for the HL and LL groups with and without global signal regression. The imaging data were reprocessed without regressing the global signal. Focusing on insula-amygdala connectivity, we observed a medium effect size of Cohen’s D=0.62, suggesting that a group difference is not solely a function of global signal regression. This effect size was lower than the effect size observed with global signal regression (Cohen’s D=1.58).


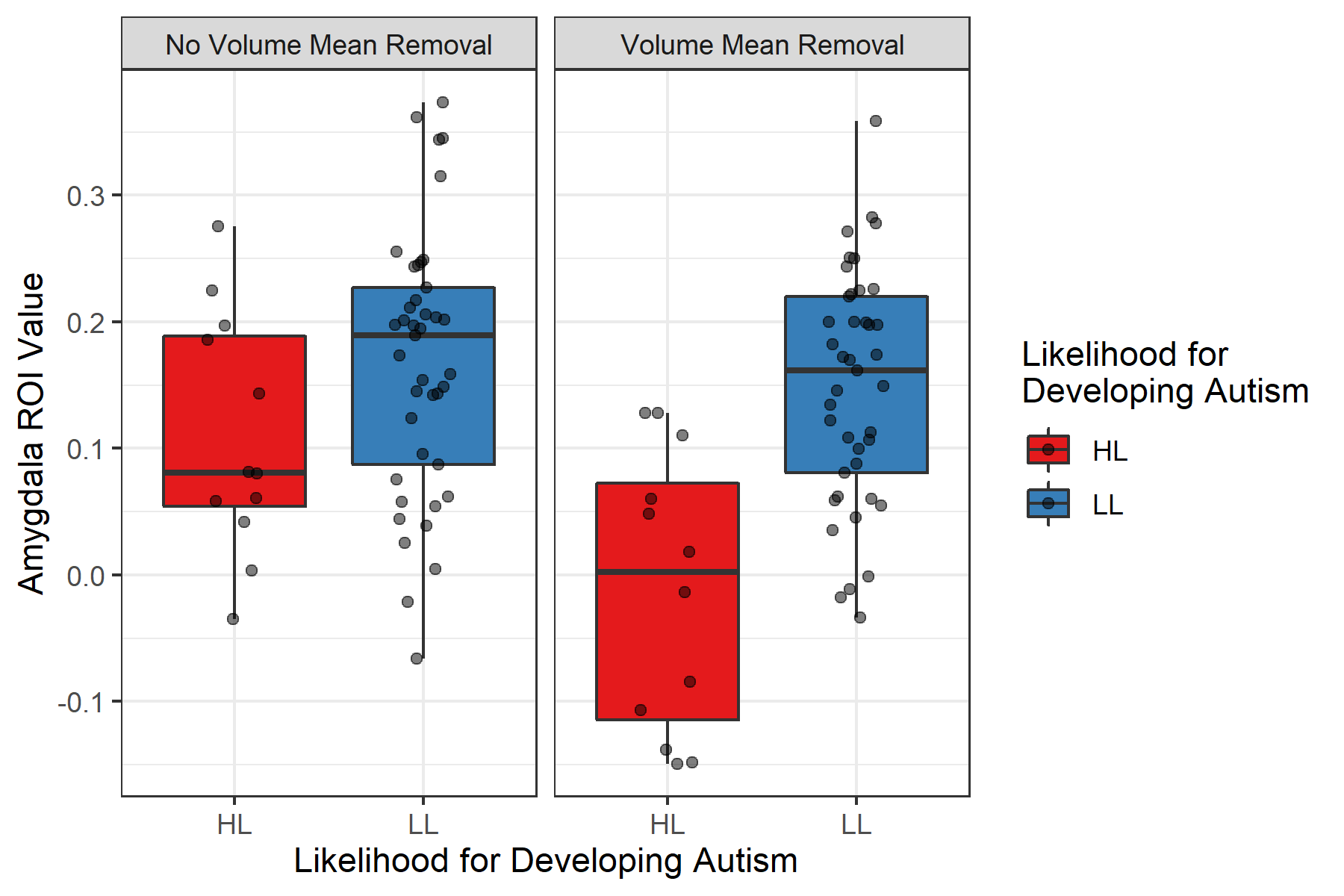


Exploration into sex differences in the HL and LL samples. Considering significant differences in sex ratios in the HL and LL groups, we extracted left aINS – left amygdala connectivity values and conducted a post-hoc group x sex ANOVA with sex as a factor, with PMA as a covariate. The ANOVA indicated a significant effect of group, F(1, 48) = 26.68, p<.001, no effect of sex, F (1, 48) = .38, p=.539, and a significant group x sex interaction, F (1, 48) = 4.28, p=.044. The contribution of PMA to the model was negligible, F (1, 48) = .09, p=.761. Post-hoc comparisons with Tukey correction for multiple comparisons indicated that while HL females (M=0.12, SD=0.01) had statistically comparable left aINS – left amygdala connectivity to LL females (M=0.15, SD=0.09) (Cohen’s d = .468, p=.981) and LL males (M=0.15, SD=0.10) (Cohen’s d = .422, p=.972), the HL males (M=-0.04, SD=0.10) showed left aINS – left amygdala hypoconnectivity compared to LL females (Cohen’s d = 2.00, p<.001) and LL males (Cohen’s d = 1.90, p<.0001) (**Figure S3**). There were no differences between LL males and LL females (p=.997), and although HL females tended to have higher left aINS – left amygdala connectivity than HL males, the contrast did not survive correction for multiple comparisons (p=.151; uncorrected: p=.036). The results suggest that the observed group differences in the aINS – amygdala connectivity are not likely to be driven by a differential composition of the samples. However, given the trends observed in the HL group, future studies need to consider sex as a key factor when examining early brain development in children with autism as well as their non-autistic siblings.

**Figure S3.** Individual left anterior insula – left amygdala functional connectivity in male (blue) and female (red) neonates with high (HL) and low likelihood (LL) for developing autism. Small dots represent individual data points, large dots represent group averages, and the vertical bars represent 95% confidence intervals.


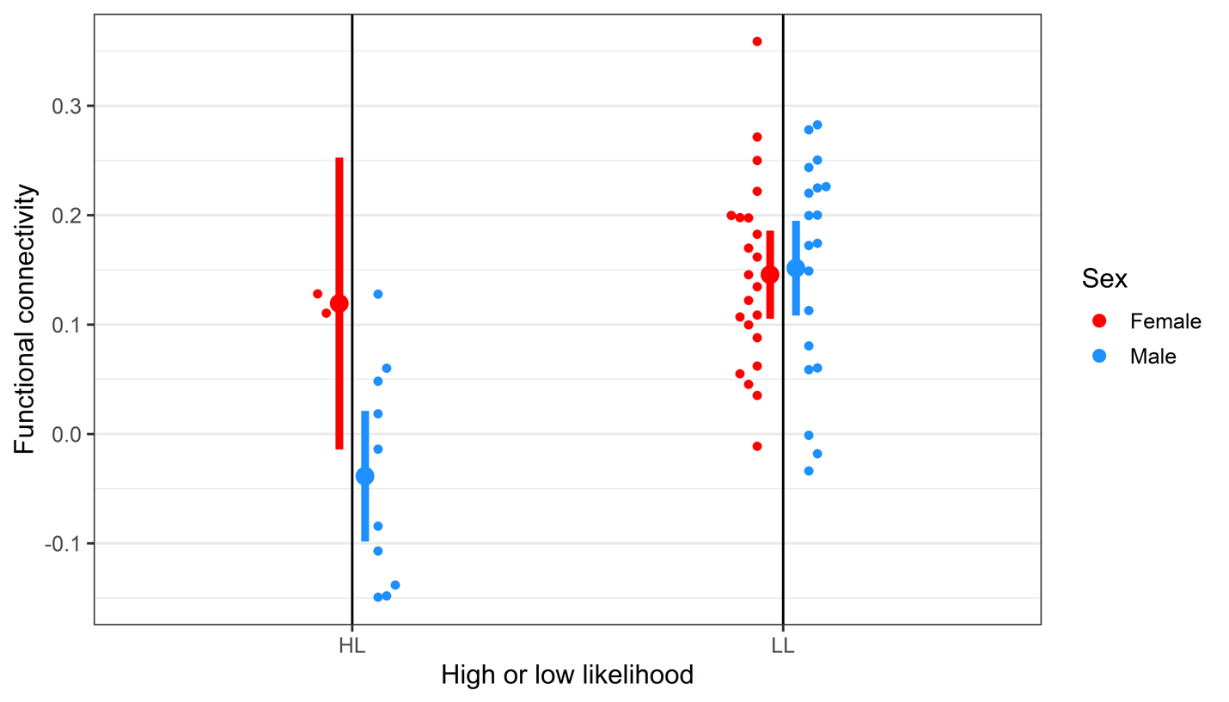

Supplement: Supplementary file 1 — Supplementary Figures. [file 41598_2022_20617_MOESM1_ESM.docx]
